# Cadaveric study of the ultrasound-guided erector spinae plane block over the dorsolateral edge of the transverse process of the ninth thoracic vertebra in dogs: transverse vs. longitudinal approach

**DOI:** 10.3389/fvets.2026.1959546

**Published:** 2026-10-13

**Authors:** Manuel Enrique Herrera-Linares, Sandra Sanchis-Mora, Carla Zamora-Perarnau, Nuria Fernández-Salesa, Bartolomé Rico-Pérez, Manuel Enrique Herrera-Gutiérrez, Claudio Iván Serra-Aguado, Rocío Fernández-Parra

**Affiliations:** 1 Doctoral School, Catholic University of Valencia San Vicente Mártir, Valencia, Spain; 2 Anesthesia Service, Pride Veterinary Referrals, Scarsdalevets, Inspiring Vet Care, Derby, United Kingdom; 3 Department of Small Animal Medicine and Surgery, Faculty of Veterinary Medicine and Experimental Sciences, Catholic University of Valencia San Vicente Mártir, Valencia, Spain; 4 Anaesthesia Service, Anicura San Vicente Veterinary Hospital, Alicante, Spain; 5 Veterinary Referral Hospital UCV, Catholic University of Valencia San Vicente Mártir, Valencia, Spain; 6 Surgery Service, FHCV-UAB, Facultat de Veterinària de la UAB, Universitat Autònoma de Barcelona, Barcelona, Spain; 7 Anesthesia Service, Southfields Veterinary Specialists, Basildon, United Kingdom; 8 Department of Intensive Care Medicine, Regional University Hospital of Málaga, Málaga, Spain; 9 Department of Medicine and Dermatology, Faculty of Medicine, University of Málaga, Málaga, Spain

**Keywords:** analgesia, anatomy, canine, interfascial plane, regional anesthesia, thorax

## Abstract

**Introduction:**

Studies of the ultrasound-guided thoracic erector spinae plane (ESP) blocks in dogs are limited. This study aimed to describe a transverse (TV) ultrasound-guided ESP approach at the dorsolateral edge of the transverse process (DLETP) of T9, compare its distribution with a longitudinal (LNG) approach, and analyze the thoracic transverse process anatomy.

**Methods:**

Twenty-three canine cadavers were used in this double-blinded experimental study. An ESP injection using 0.6 mL/kg of dye/contrast was performed on each lateral of 21 cadavers with a TV or LNG approach at T9, followed by computed tomography and dissection.

**Results:**

Five cadavers were excluded from dissection analysis. All TV injections were placed over the DLETP, whereas 18.75% of LNG injections were misplaced laterally. Both approaches produced craniocaudal spread within the ESP, with median (range) spreads of 8 (5–14) and 8.5 (4–13) vertebrae for the TV and LNG groups, respectively. Cranial spread exceeded caudal in the TV group, with 4.5 (2–6) vs. 3.5 (0–7) vertebrae (*p* = 0.027), and LNG, with 5 (3–7) vs. 3 (0–6) (*p* = 0.004). Staining of the medial branches of the dorsal rami (MBDR) of the spinal nerves showed median (range) values of 5 (4–8) and 5 (0–6) in TV and LNG. Cranial MBDR staining predominated in TV, with 4.5 (1–6) over caudal with 1 (0–3) (*p* = 0.003), and LNG, with 4 (0–6) vs. 0 (0–3) (*p* < 0.001). Lateral branches (LBDR) staining showed 4 (1–9) and 4 (2–12) in TV and LNG. Cranial LBDR staining exceeded caudal in TV, with 3 (0–6) vs. 1 (0–3) (*p* = 0.019), and LNG, with 3 (1–8) vs. 1 (0–5) (*p* = 0.034). Ventral branches staining occurred in 10/16 TV injections (62.5%) and 11/16 (68.75%) LNG. Both approaches showed epidural and sympathetic trunk spread while one intravascular injection occurred in LNG. No significant differences were found between approaches. The DLETP was predominantly absent caudal to T10 and smaller than the transverse process (*p* < 0.001).

**Conclusion:**

The TV and LNG approaches produced comparable nerve staining and ESP spread at T9 in dogs, with preferential cranial distribution. The TV approach reduced injectate misplacement. The DLETP was smaller than the transverse process and consistently identified cranial to T11.

## 1 Introduction

Since the erector spinae plane (ESP) block was first described in humans (1), a growing interest in this technique has emerged in both veterinary and human medicine, with cadaveric studies conducted across several animal species (2–12).

Various theories regarding the mechanism of action of the ESP block have been proposed in humans (8, 13). The most consistently observed pattern involves dye spread to the lateral (LBDR) and medial branches (MBDR) of the dorsal rami of the spinal nerves. These nerves innervate the dorsolateral aspect of the spine and have been consistently stained in canine cadavers (2, 4). Further spread to the ventral branches of the spinal nerves (VBSN), paravertebral space, sympathetic trunk, epidural space, and lymphatic system has been reported in both humans and veterinary species (3, 12, 14–18).

The erector spinae muscles form part of the epaxial musculature covering the dorsolateral aspect of the vertebrae and ribs, including the *longissimus, semispinalis*, and *iliocostalis* muscles (4). The ESP block was originally performed using an ultrasound (US)-guided longitudinal parasagittal approach (LNG) (1, 2, 4). More recently, a transverse (TV) approach has been described in both human and veterinary medicine (18–21). To date, only one veterinary study has directly compared both approaches over the same area, the transverse process of T12, finding comparable injectate spread (18). However, these results cannot be extrapolated to more cranial vertebrae due to differences in musculature and vertebral anatomy (22, 23).

The dorsolateral edge of the transverse process (DLETP), the insertion site of the erector spinae and *multifidus* muscles, was the anatomical landmark originally described for performing the ESP block at T9 level in dogs (4). However, this landmark is not consistently identifiable across all thoracic vertebrae (18), and its presence, morphology, and variability have not been comprehensively characterized.

The objectives of this study were to describe a novel US-guided TV approach for the thoracic ESP block over the DLETP, to compare nerve staining and injectate spread between the LNG and the TV US-guided ESP block approaches at T9, and to study the anatomy of the transverse process of the thoracic vertebrae. We hypothesized that both approaches would produce similar nerve spread and dye distribution.

## 2 Materials and methods

This prospective, randomized, double-blinded, experimental and cadaveric anatomical study has been performed under the ethical approval of the Catholic University of Valencia (CEEAUCV2210).

### 2.1 Animals

A total of 23 adult canine cadavers were included in this study and allocated into two separate weeks (Figure 1). Twelve cadavers were used in the first week and 11 cadavers in the second week (Figure 1). All cadavers were thawed at room temperature for 48 h (20–22 °C). Two cadavers out of the 23, one for each week, were randomly allocated to a preliminary anatomical investigation. The remaining 21 were used to compare the injectate spread between traditional LNG approach with the TV approach at T9 and to analyze the anatomy of the transverse process of the thoracic vertebrae (Figure 1). The number of cadavers was based on similar previously published cadaveric studies (4, 18, 24–26). A larger number of cadavers than in previous studies was selected to improve the precision and robustness of the anatomical observations, and to account for potential exclusions. Exclusion criteria included spinal trauma, any damage that could affect permeability of the ESP and excessive accumulation of gas within the ESP and muscles due to putrefaction. All specimens were obtained after natural death or euthanasia for reasons unrelated to this study. The cadavers from the first experimental week were also used in an unrelated cadaveric study, without interference with the anatomical structures evaluated in the present investigation (25).

**Figure 1:**
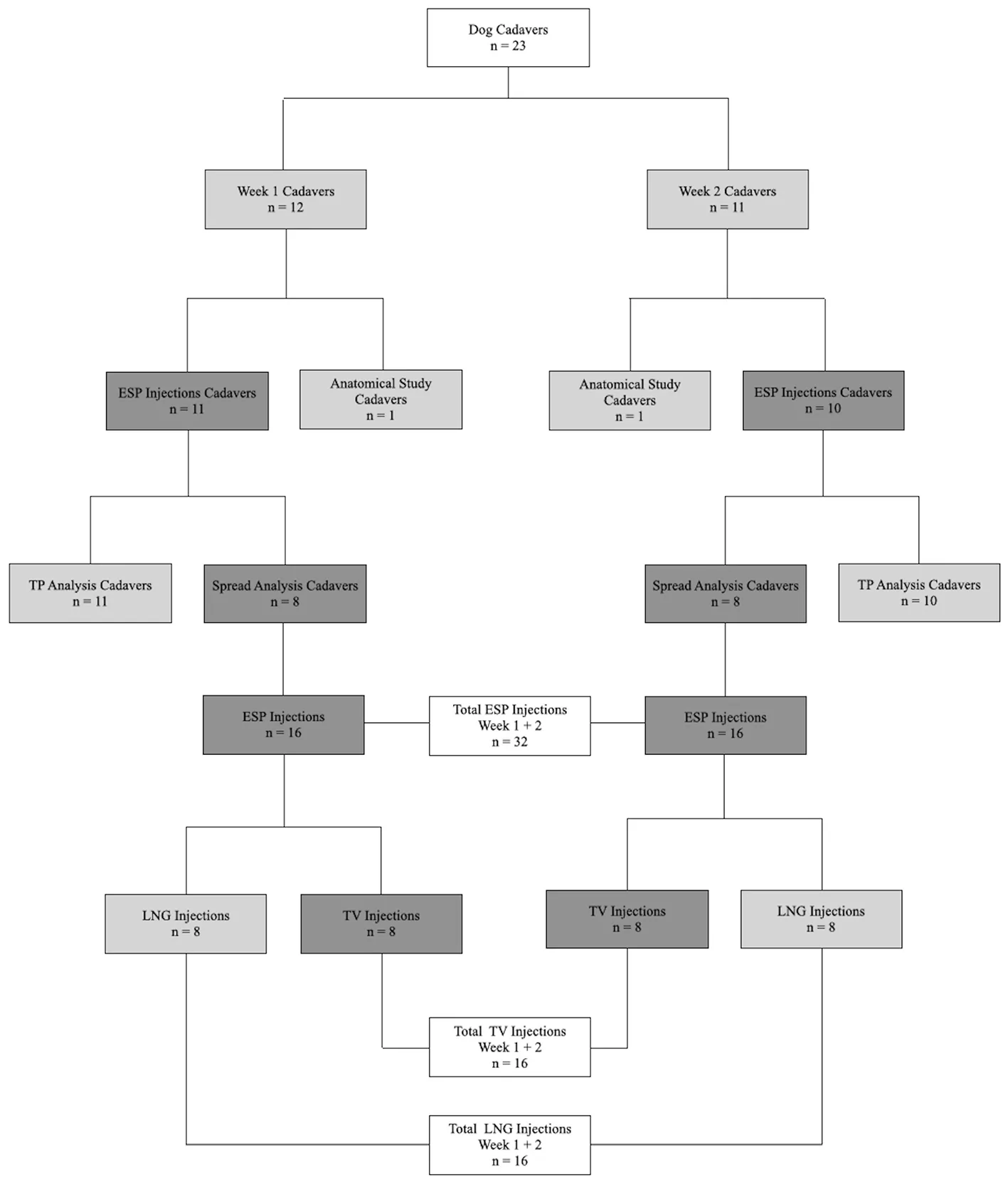
Diagram illustrating cadaver allocation and distribution of the erector spinae plane injections during the study. ESP, erector spinae plane; LNG, longitudinal approach; TP, transverse process; TV, transverse approach.

### 2.2 Preliminary anatomical assessment

One of the 23 cadavers was allocated each week (two in total) to a preliminary anatomical assessment and underwent ultrasonographic examination followed by gross anatomical dissection to familiarize the investigators with the regional anatomy and technique (Figure 1). With the animal positioned in sternal recumbency, the dorsal thoracic region was clipped. A veterinary anesthetist performed the ultrasonographic evaluation using a 16–3 MHz linear transducer connected to a Samsung HM70EVO portable US machine (Samsung^®^) to identify the relevant landmarks for the TV approach for the ESP injection. Gross anatomical dissection was then performed following the protocol described by Herrera-Linares et al. (18). A different veterinary surgeon performed the dissections during each experimental week to identify the medial, lateral, and ventral branches of the spinal nerves, as well as the paravertebral space and the sympathetic trunk (Figure 2a).

**Figure 2:**
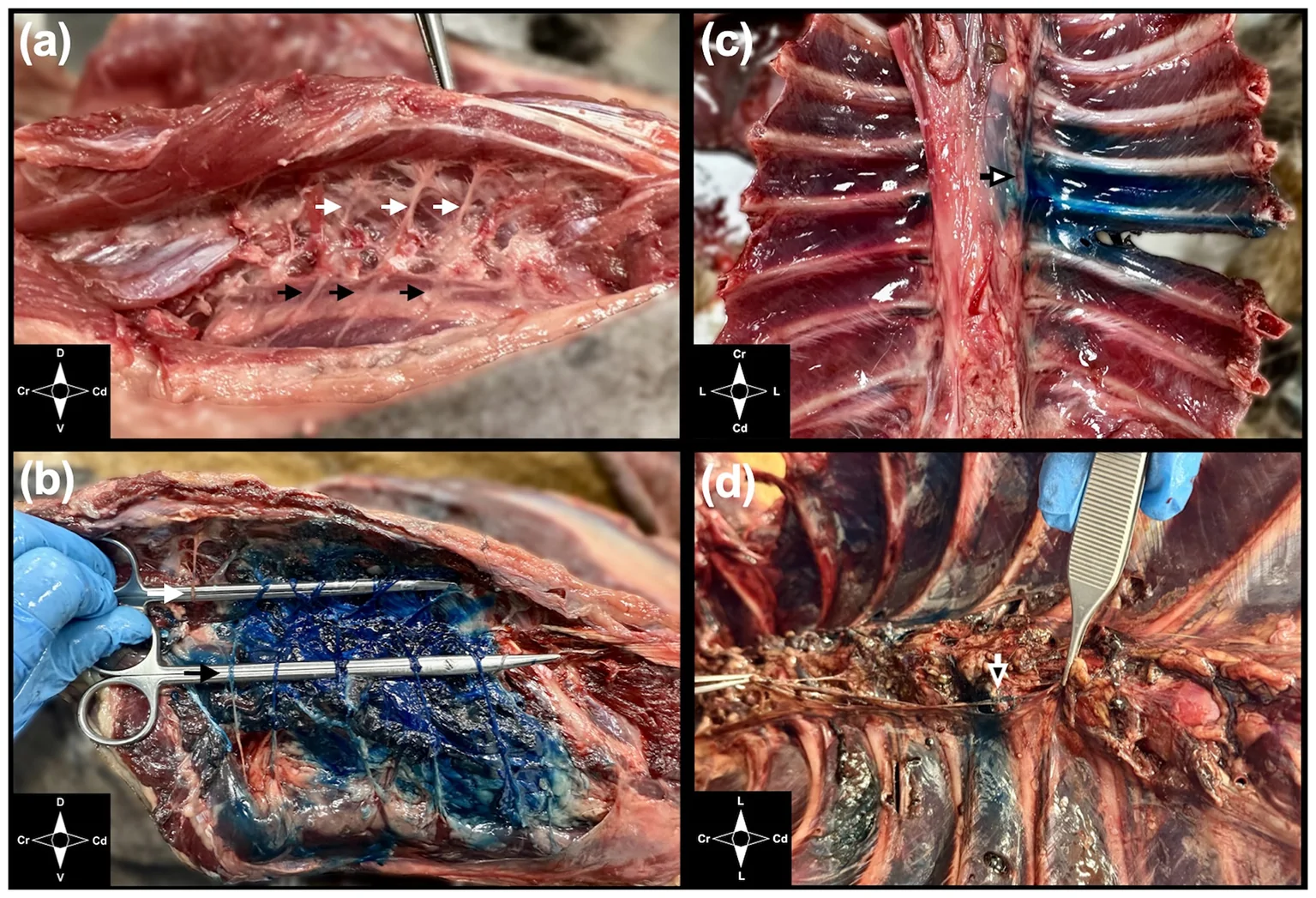
Images of the anatomical dissection. **(a)** Lateral views showing the erector spinae plane (ESP) with the identification of the medial (white arrow) and lateral (black arrow) branches of the dorsal rami of the spinal nerves. **(b–d)** Anatomical dissection following ESP injection using a methylene blue and iohexol contrast solution. **(b)** Lateral view with dye of the medial (white arrow) and lateral (black arrow) branches of the dorsal rami of the spinal nerves. **(c, d)** Ventral thoracic view following removal of the ventral and lateral attachments of the diaphragm, sternum, and thoracic and abdominal viscera showing: **(c)** paravertebral spread (black arrow with white center); **(d)** sympathetic trunk dye (white arrow with black center). Cd, caudal; Cr, cranial; D, dorsal; L, lateral; V, ventral.

### 2.3 Ultrasound-guided LNG & TV ESP injection

Out of the 23 thawed dogs, 11 and 10 cadavers underwent a US-guided ESP injection during the first and second week, respectively (Figure 1). Each cadaver received a LNG and a TV ESP injection in each lateral. The injection sites (left or right lateral) and the different approaches (LNG or TV) were randomly assigned using a randomization software (https://www.randomizer.org) for all the 21 treated dogs. In the first experimental week, all LNG injections were performed by one veterinary anesthetist, and all TV injections were performed by a different veterinary anesthetist. In the second week, because the anesthetist who performed the LNG injections during the first week was unavailable, all injections were performed by the veterinary anesthetist responsible for the TV injections in the first week. All cadavers were positioned in sternal recumbency, and the injection area was clipped prior to the procedure. A 16–3 MHz linear transducer connected to a Samsung HM70EVO portable US machine (Samsung^®^) was used to guide all injections.

For the LNG approach, the linear transducer was placed parallel to the spine on the ipsilateral side of the injection, immediately caudal to the last rib. The probe was moved cranially until the bony shadow of the last rib was identified, and this process was repeated cranially until the 9th rib was visualized. At this level, the transducer was moved medially to obtain the irregular hyperechoic shape corresponding to the DLETP of T9 (Figures 3b, c). Using an in-plane technique, an echogenic insulated needle (22-gauge, 80 mm, Ultraplex 360^®^ B. Braun Medical Ltd.) was advanced through the erector spinae musculature in a cranial caudal direction until the tip contacted the dorsal aspect of the DLETP (Figures 3a, c) (4, 18, 24). The needle tip was gently pressed against the bone and a total volume of 0.6 mL/kg of a 1:1 mixture of methylene blue (Methylthioninium Chloride Injection 1% w/v; Martindale Pharmaceuticals Ltd.) and iohexol contrast medium (Omnipaque 300; GE Healthcare) was injected as rapidly as possible.

**Figure 3:**
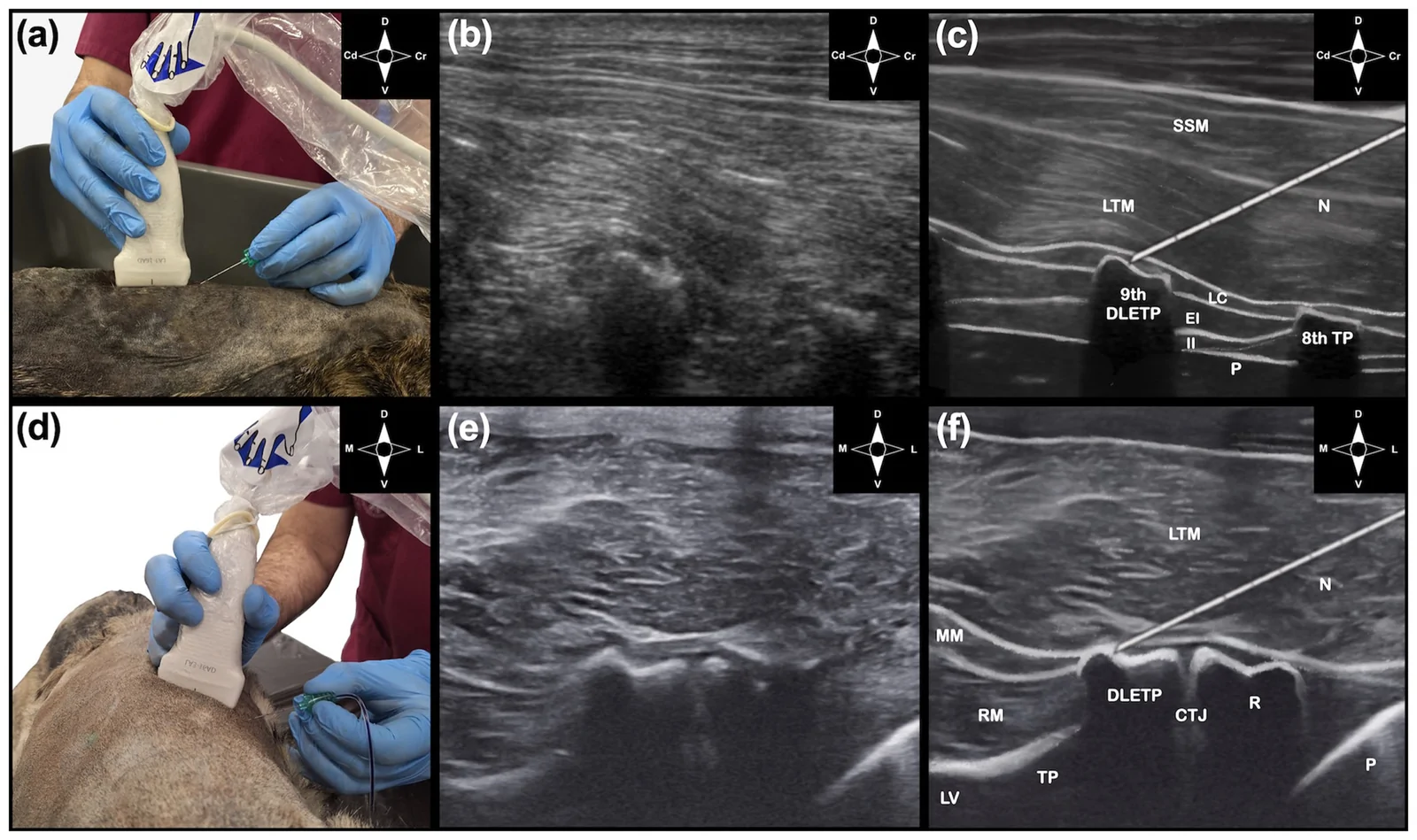
Ultrasound-guided erector spinae plane (ESP) injection at T9. Technique, images, and landmarks of the longitudinal (LNG) and transverse (TV) approaches. **(a)** External view of the LNG approach for the ESP injection, showing ultrasound (US) transducer orientation and needle direction. **(b)** Ultrasound image of the LNG ESP approach. **(c)** Schematic representation of **(b)** with the needle in place. **(d)** External view of the TV approach of the ESP injection with the orientation of the US transducer and the needle direction. **(e)** Ultrasound image of the TV ESP approach. **(f)** Schematic representation of **(e)** with the needle in place. Cd, caudal; Cr, cranial; CTJ, costotransverse joint; D, dorsal; DLETP, dorsolateral edge of the transverse process; EI, external intercostal muscle; II, internal intercostal muscle; L, lateral; LC, *levatores costarum* muscle; LTM, *longissimus thoracis* muscle; LV, lamina of the vertebra; M, medial; MM, *multifidus* muscle; N, needle; P, pleura; R, rib tubercle; RM, *rotatores* muscles; SSM, *semispinalis* muscle; TP, transverse process; V, ventral.

For the TV approach, the transducer was placed caudal to the last rib at the midline, perpendicular to the spine, and slightly tilted toward the side of the injection. The probe was then moved cranially until the rounded bony shadow of the 13th rib appeared on the lateral aspect of the image. From this point, the rib was traced in a dorsolateral-to-ventromedial direction until it contacted the hyperechoic surface of the corresponding transverse process that appeared medially. This procedure was repeated cranially until the 9th rib was visualized contacting the transverse process of T9, placing the ninth costotransverse joint within the imaging window (Figures 3e, f). This positioning allowed clear visualization of the lamina, transverse process, DLETP, costotransverse joint, and rib tubercle, thereby facilitating precise identification of the injection site (Figures 3e, f). Using an in-plane technique, the needle was advanced in a dorsolateral-to-ventromedial direction until the tip reached the dorsal aspect of the DLETP (Figures 3d, f; Supplementary video S1). The needle tip was gently pressed against the bone and the same dye/contrast mixture previously used was then injected at this location at the same rate and with the same total volume previously described for the LNG approach.

### 2.4 Computed tomography study & anatomical dissection

Immediately after the injections, each cadaver was transferred to the computed tomography (CT) area. A thoracolumbar CT scan was performed using a 16–slice multidetector CT scanner (Siemens Somatom Scope, Munich, Germany) in helical mode. CT acquisition parameters included a 512 × 512 matrix, a pitch of 0.9, 1.0–mm slice thickness, 180 mAs, 130 kV, and a display field of view adjusted to patient size. Images were reviewed using a DICOM viewer (OsiriX MD v14.2.0) with soft tissue (window width [WW] = 350 HU, window level [WL] = 40 HU) and bone (WW = 1500 HU, WL = 450 HU) reconstruction algorithms. The scans were evaluated by the same veterinary radiologist blinded to the treatment group. The length of the transverse process, presence and length of DLETP, and presence of transitional vertebrae or ribs or vertebrae abnormalities were recorded (Figure 4). The extent of injectate spread within the ESP, epidural space, paravertebral space, and lymphatic system was measured and compared between the two approaches. A semiquantitative scoring system was used to assess the degree of gas produced by putrefaction, using the presence of gas within the erector spinae plane and surrounding musculature as a reference on the CT images. Scores were defined as follows: 0 = no gas detected, 1 = moderate but acceptable presence of gas, and 2 = excessive and unacceptable presence of gas (Figure 5).

**Figure 4:**
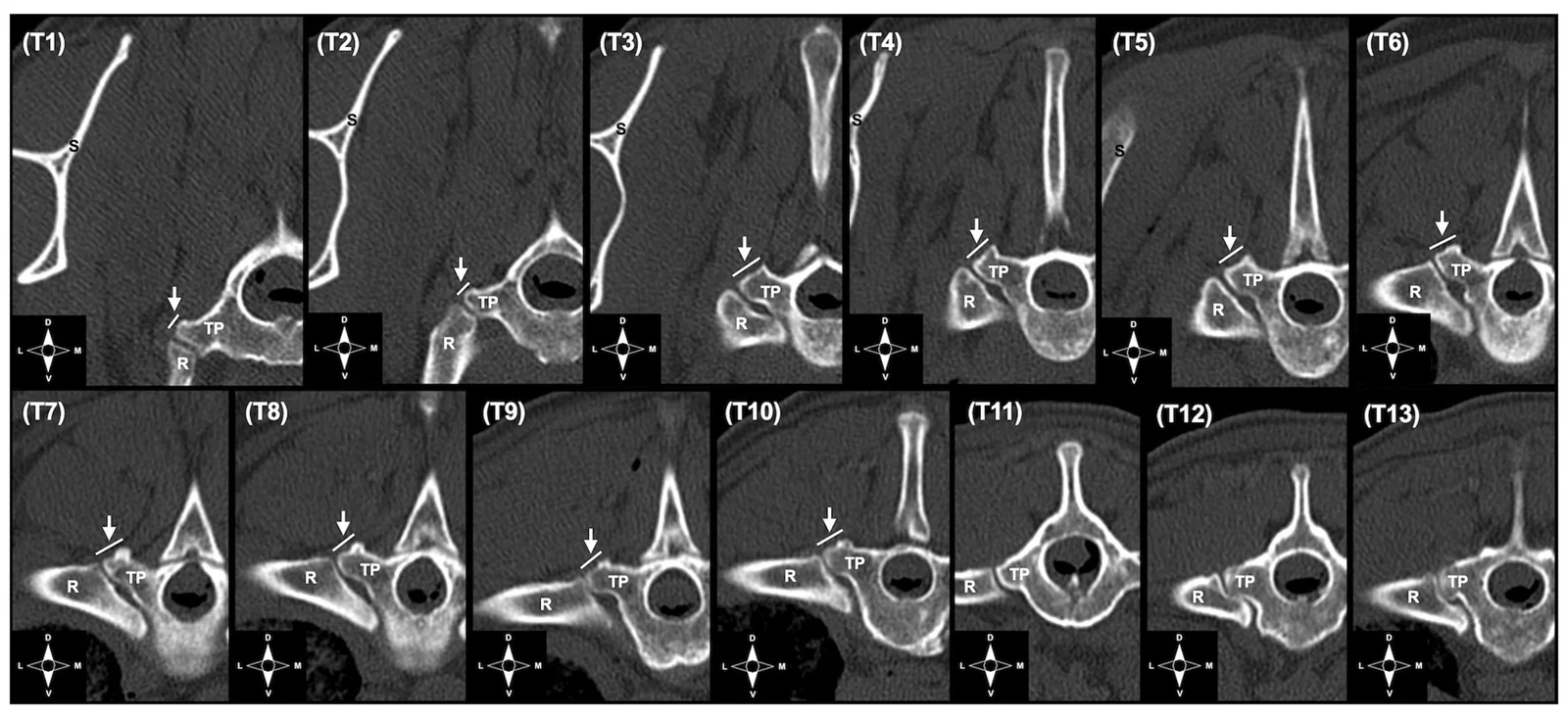
Transverse CT scans from T1 to T13 showing the transverse process and the dorsolateral edge of the transverse process (white arrow) with its length (white line). D, dorsal; L, lateral; M, medial; R, rib; S, scapula; TP, transverse process; V, ventral.

**Figure 5:**
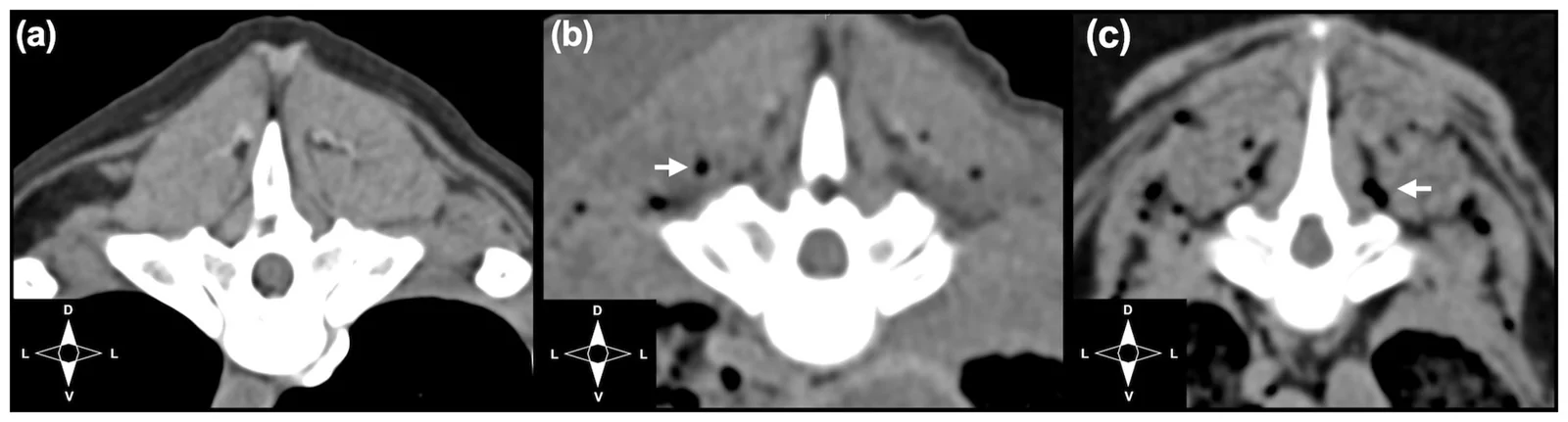
Representative transverse CT images illustrating the semiquantitative scoring system used to assess postmortem gas production associated with putrefaction within the erector spinae plane and surrounding musculature. **(a)** Score 0: no gas detected. **(b)** Score 1: moderate but acceptable presence of gas (white arrow). **(c)** Score 2: excessive and unacceptable presence of gas (white arrow).

All anatomical dissections were performed immediately after CT by the same surgeon involved in the preliminary anatomical study, following the same technique as previously used (18). The surgeons were blinded to treatment allocation. Staining of the MBDR, LBDR, and VBSN was considered successful when dye circumferentially surrounded the nerve over a length greater than 1 cm (27). The longitudinal extent of spread within the ESP was also recorded by counting the number of vertebrae of the spread. Vertebral spread was considered complete if at least 50% of its length was covered (28).

### 2.5 Statistical analysis

Results are presented as *n* (%) for categorical variables, median (range) for non-parametric continuous data, and mean ± standard deviation (SD) for parametric data. Normality of the analyzed variables was assessed using the Kolmogorov–Smirnov test. Categorical variables were compared using Fisher's exact test. For continuous variables, independent-samples comparisons were made using Student's *t*-test (parametric data) or the Mann-Whitney U test (non-parametric data), while paired comparisons were made using Wilcoxon signed-rank test (non-parametric data). For comparisons of continuous variables involving more than two groups, a one-way ANOVA was used, followed by Tukey's *post hoc* test for comparisons. A *p*-value < 0.05 was considered statistically significant for all tests. Hodges-Lehmann median difference 95% confidence interval (CI) was calculated for statistically significant differences in both independent and paired samples. For data analysis and figure generation, IBM SPSS Statistics for macOS (Version 30.0; IBM Corp.) and GraphPad Prism (Version 11) were used.

## 3 Results

### 3.1 Animals

Twenty-one dogs received a bilateral ESP injection. Cadavers included 4 mixed-breed dogs (19.05%), 3 Greyhounds (14.29%), 3 Beagles (14.29%), 2 Border Collies (9.52%), and 1 each of Bichon Frise, Braco, Cocker Spaniel, German Shepherd, Husky, Miniature Collie, Podenco, Poodle, and Staffordshire Terrier (4.76% each). The mean (± SD) body weight was 17.69 ± 6.24 kg with a body condition score of 6 (5–8) out of 9.

After CT evaluation, 1 of the 21 cadavers (4.76%) was excluded for the analysis of the injectate spread due to multiple foreign bodies within the musculature that could potentially affect ESP permeability. In the remaining 20 cadavers, the semiquantitative assessment of gas accumulation due to putrefaction yielded a score of 0 in 9 cadavers (45%), 1 in 7 cadavers (35%), and 2 in 4 cadavers (20%) out of 20. The 4 cadavers (20%) with a score of 2 were excluded for analysis of the spread due to excessive gas accumulation within the ESP and surrounding musculature (Figure 5). CT evaluation also revealed anatomical abnormalities among the 21 cadavers: 2 out of 21 (9.52%) had a transitional thoracolumbar vertebra, 1 (4.76%) exhibited bone neoformation of the ventral lamina caudal to T10, 5 (23.81%) presented spondylosis, and 1 (4.76%) had intervertebral disc disease. All the 21 cadavers were included for the anatomical study of the transverse process (Figure 4).

### 3.2 Ultrasound-guided ESP injections & groups

Each cadaver received both, a TV and an LNG injection on each side, resulting in a total of 21 injections per group (TV and LNG) and 42 injections overall. After applying the previously described exclusion criteria following CT evaluation, 32 of the total 42 injections (76.19%) were included in the analysis of spread, leaving 16 injections per group (Figure 1). All injections in the TV group were performed over the intended target, the DLETP at T9. In contrast, 3 injections out of the 16 (18.75%) in the LNG group, 1 by a veterinary anesthetist and 2 by the other, were inadvertently placed lateral to the DLETP resulting in predominantly ventrolateral spread (Figures 6b, d).

**Figure 6:**
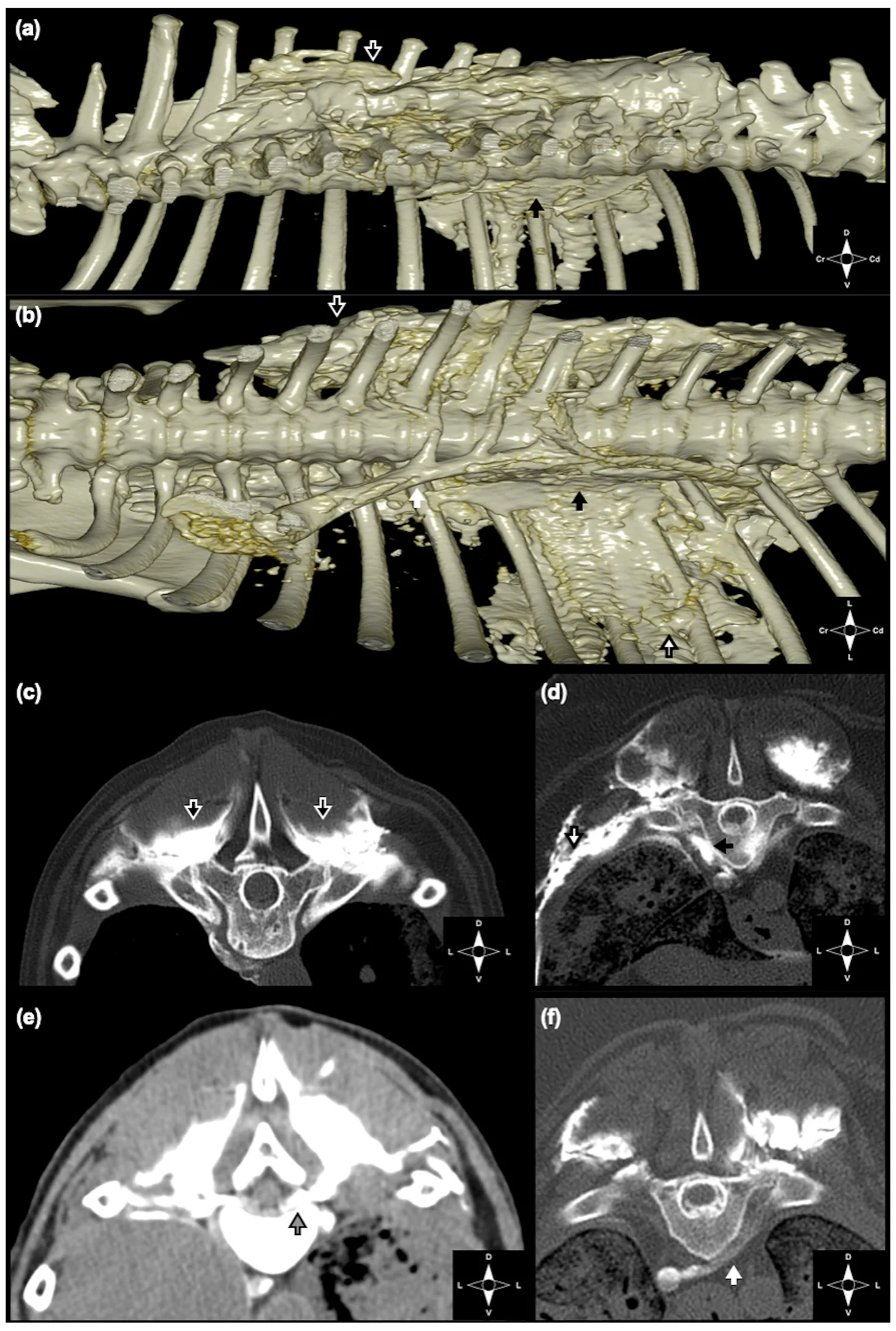
Computed tomography (CT) imaging study showing distribution of contrast/dye solution following the erector spinae plane (ESP) injection. **(a, b)** Three-dimensional CT volume renderings showing: **(a)** lateral view with spread within the ESP (white arrow with black center) and paravertebral spread (black arrow); **(b)** ventral view with spread within the ESP (white arrow with black center), paravertebral spread (black arrow), intravascular spread (white arrow) and lateral spread outside the ESP (black arrow with white center). **(c–f)** Transverse CT images showing: **(c)** spread within the ESP (white arrow with black center); **(d)** paravertebral spread (black arrow) and lateral spread outside the ESP (black arrow with white center); **(e)** epidural spread (black arrow with grey centre); **(f)** intravascular spread (white arrow). Cd, caudal; Cr, cranial; D, dorsal; L, lateral; V, ventral.

### 3.3 Dissection analysis of injectate spread

Dissection-recorded total spread length into the ESP was 8 (5–14) and 8.5 (4–13) vertebrae for the TV and LNG groups, respectively. The most cranial spread reached T3 in the TV group and T2 in the LNG group, whereas the most caudal spread reached L3 and L2, respectively. Cranial spread was found in 16/16 (100%) in both groups while caudal was present in 14/16 (87.5%) in the TV group and 12/16 (75%) in LNG. Cranial spread was significantly greater than caudal spread in both the TV group with 4.5 (2–6) vs. 3.5 (0–7) vertebrae (*p* = 0.027, 95% CI: −2.5, 0), and the LNG group with 5 (3–7) vs. 3 (0–6) vertebrae (*p* = 0.004, 95% CI: −3, −1) (Figure 7a).

**Figure 7:**
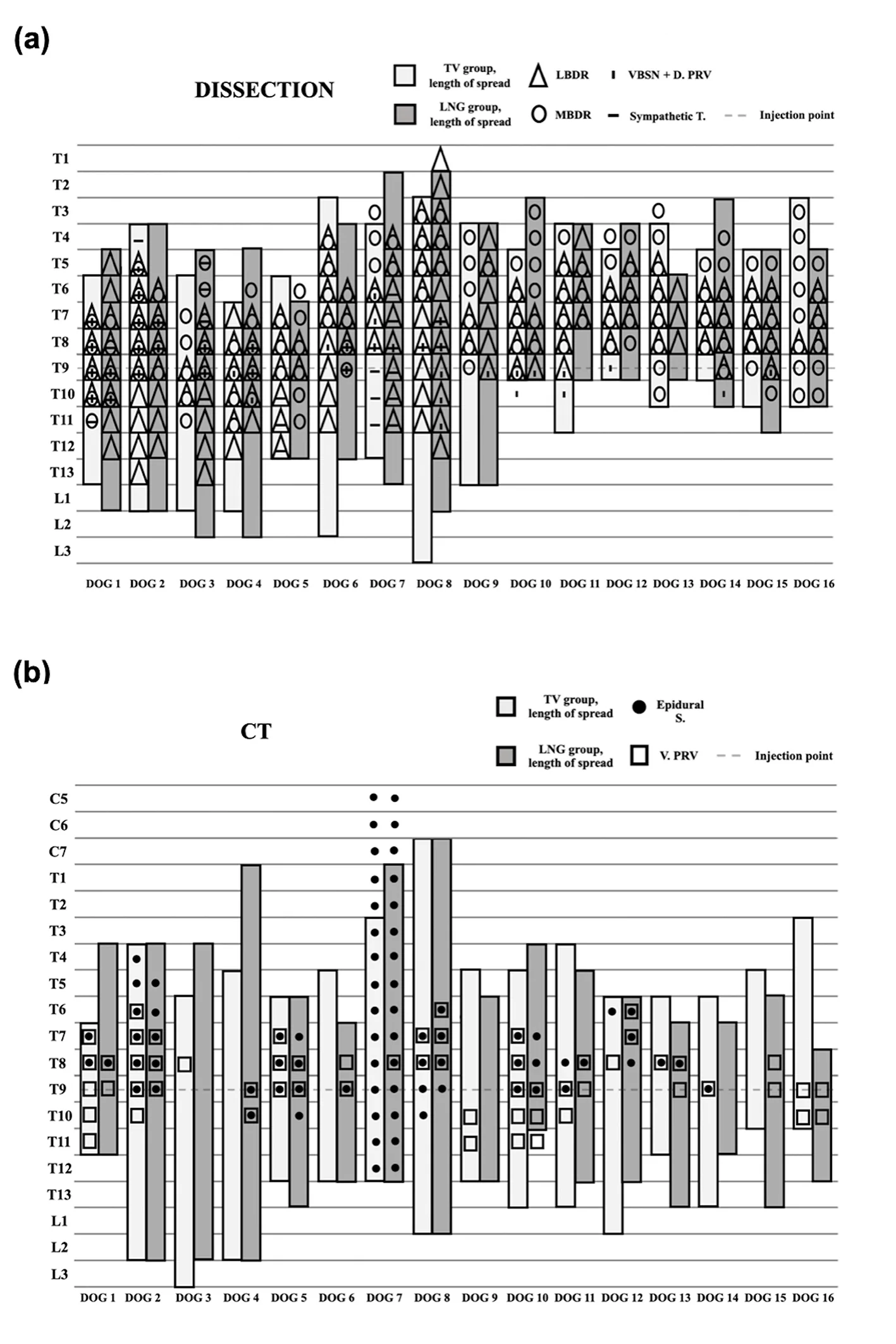
Spread following transverse (TV) and longitudinal (LNG) approaches of the erector spinae plane (ESP) injections in each cadaver. **(a)** Dissection spread within ESP including dye of the lateral branches of the dorsal rami of the spinal nerves (LBDR), medial branches of the dorsal rami of the spinal nerves (MBDR), and ventral branches of the spinal nerves (VBSN), as well as spread into the dorsal paravertebral space (D. PRV), and over the sympathetic trunk (Sympathetic T.). **(b)** Computed tomography (CT) spread within the ESP, epidural space (Epidural S.), and ventral paravertebral space (V. PRV). C, cervical vertebra; L, lumbar vertebra; T, thoracic vertebra.

MBDR were stained in all injections (100%) in the TV group and in 15 of 16 injections (93.75%) in the LNG group, with a median (range) of 5 (4–8) and 5 (0–6) nerves stained, respectively (Figures 2b, 7a). The most cranial and caudal MBDR stained were T3 and T11 in both groups. In the TV group, cranial staining was present in 16/16 (100%) injections with a median of 4.5 (1–6) MBDR stained, compared with 13/16 (81.25%) injections and 1 (0–3) caudally. In the LNG group, cranial staining was present in 15/16 (93.75%) injections with a median of 4 (0–6) branches stained, compared with 11/16 (68.75%) injections and a median of 0 (0–3) branches caudally. Cranial staining was significantly more extensive than caudal in both groups (TV: *p* = 0.003, 95% CI: −4.5, −1.5); LNG: *p* < 0.001, 95% CI: −4, −2).

LBDR were stained in all injections (100%) for both approaches, with a median (range) of 4 (1–9) and 4 (2–12) branches stained for the TV and LNG groups, respectively (Figures 2b, 7a). Across all cadavers, the most cranial LBDR stained was T3 in the TV group and T1 in the LNG group, while the most caudal was T13 in both groups. Cranial staining was present in 14/16 (87.5%) injections in the TV group and 16/16 (100%) in the LNG group, with a median of 3 (0–6) and 3 (1–8) branches stained, respectively. Caudal staining occurred in 14/16 (87.5%) injections in the TV group and 13/16 (81.25%) in the LNG group, with a median of 1 (0–3) and 1 (0–5) branches stained in the TV and LNG group, respectively. Cranial spread of LBDR also significantly exceeded caudal spread in the TV (*p* = 0.019, 95% CI: −3.5, −0.5) and the LNG group (*p* = 0.034, 95% CI: −3, 0).

Complete staining of the VBSN within the dorsal paravertebral compartment was observed in 10/16 injections (62.5%) in the TV group and 11/16 injections (68.75%) in the LNG group, with a median of 1 (0–5) VBSN stained in both groups (Figures 2c, 7a). Cranial staining of the VBSN was present in 5/16 (31.25%) injections in the TV group and 6/16 (37.5%) in the LNG group while caudal staining was observed in 4/16 (25%) injections in the TV group and 7/16 (43.75%) in the LNG group. Ventral paravertebral spread observed during dissection occurred in 7/16 (43.75%) injections in the TV group and 6/16 (37.5%) in the LNG group (Figure 2c). Sympathetic spread was observed in 6/16 (37.5%) and 7/16 (43.75%) injections in the TV and LNG groups, respectively (Figures 2d, 7a).

Dogs 7, 9, and 13 received LNG injections misplaced lateral to the DLETP and showed either no MBDR staining or staining of only 1 or 2 MBDR, located more cranially than expected relative to the injection site (Figure 7a). A similar cranial shift was observed in dog 7 within the TV group, although CT analysis confirmed a spread pattern similar to the other injections using this approach. Dog 8 also showed a cranial shift in the level of the first stained nerve root with the TV and LNG approaches, despite CT confirming a spread pattern comparable to that observed in the remaining injections for each group (Figure 7a).

No statistically significant differences were detected between the TV and LNG groups in the spread analysis on cadaveric dissection.

### 3.4 CT analysis of injectate spread

CT-recorded total spread length within the ESP was 8 (5–15) and 8 (5–17) vertebrae for the TV and LNG, respectively, with the most cranial spread at C7 in both groups and the most caudal at L3 and L2 for TV and LNG, respectively (Figures 6a, c, 7b). Cranial spread showed a median (range) spread of 4 (2–8) and 3.5 (1–9) vertebrae for the TV and LNG group, respectively. Caudal spread presented 3 (1–7) vertebrae in the TV and 3 (1–6) in the LNG group. Ventral paravertebral spread was observed in 12 TV injections (75%) and 13 (81.25%) LNG during CT analysis, with a median spread of 1 (0–5) and 2 (0–3) vertebrae, respectively (Figures 6a, b, d, 7b). Epidural spread was recorded in 10 (62.5%) using the TV approach and 11 (68.75%) using LNG, with a median of 1 (0–15) for both (Figures 6e, 7b). Dog number 7 showed an increased epidural spread compared with the other animals, extending from T12 to C5 for both approaches. One (6.25%) partial intravascular injection was visualized in the LNG group with none in the TV (Figures 6b, f).

No statistically significant differences were detected between the TV and LNG groups or between cranial and caudal spread within each group after CT analysis.

### 3.5 Anatomical study of the transverse process

CT analysis of the presence and length of the transvers process and the DLETP are shown in the table in Figure 8 (Figures 4, 8a). The DLETP was absent caudal to T10 in 17.5 out of 21 cadavers (83.33%) and at T11 in 3.5 out of 21 cadavers (16.67%). In each vertebra where the DLETP was present, it was significantly smaller than the length of the transverse process (*p* < 0.001, 95% CIs are shown in Figure 8a) (Figures 4, 8a, b). Analysis of the DLETP between vertebrae showed significant differences among the first three vertebrae, as well as between these and the remaining vertebrae (*p* < 0.001) (Figure 8b). A progressive increase in size was also observed, ranging from 2.16 ± 0.58 at T1 to 4.13 ± 1.38 at T11 (*p* < 0.001) (Figures 8a, b). The ANOVA test also demonstrated overall significant differences in the transverse processes length between vertebrae (*p* < 0.001). However, the analysis between groups showed only differences between the last three vertebrae and the rest (Figures 8a, b).

**Figure 8:**
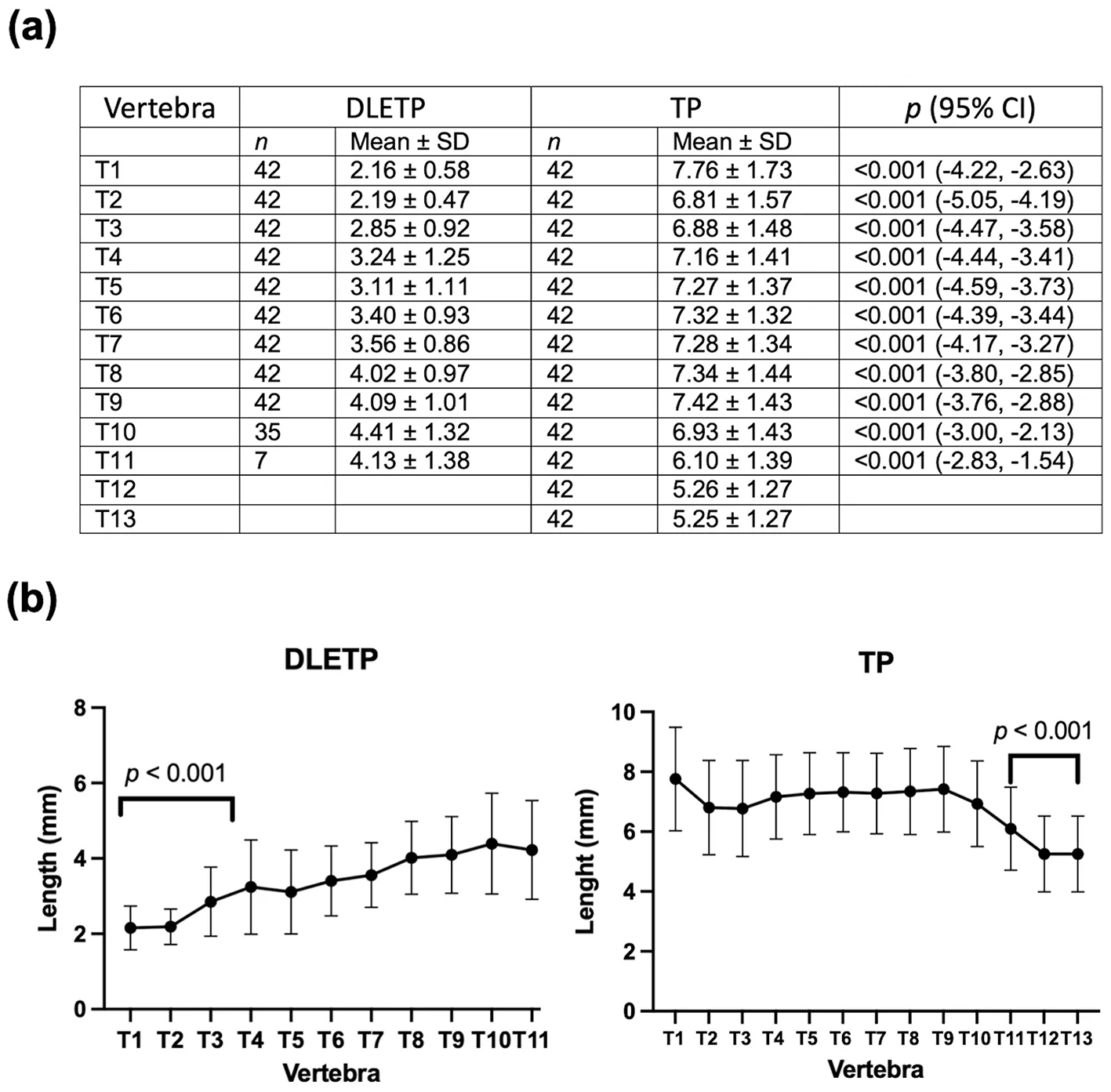
Results of the measurements of the dorsolateral edge of the transverse process (DLETP) and transverse process (TP) in the thoracic vertebrae of 21 cadavers. **(a)** Mean ± standard deviation (SD) lengths of the DLETP and TP for each thoracic vertebra, including sample size (*n*) and statistical comparisons between the two structures. **(b)** Graphical representation of the mean ± SD lengths of the DLETP and TP across the thoracic vertebral column, showing statistical comparisons between vertebrae. CI, confidence interval.

## 4 Discussion

As hypothesized, both the LNG and TV approaches using 0.6 mL/kg at T9 produced comparable spread through the ESP compartment and consistent staining of the lateral and medial branches of the dorsal rami. However, the LNG approach resulted in a greater incidence of undesired lateral spread due to misplacement of the injectate relative to the DLETP.

Following CT analysis, few animals were excluded due to excessive soft tissue gas accumulation secondary to putrefaction, which could have altered the distribution of the injected solution (29). The thawing period was selected to standardize the procedure and ensure comparability with previous canine studies (12, 18, 24). However, no comparable complications have been reported in those studies. This discrepancy may be explained by differences in ambient temperature during the thawing process, which were not consistently reported in other studies and could have introduced variability in decomposition rate (12, 18, 24). Additional factors such as body composition and cause of death may further complicate standardization. Furthermore, the absence of explicit exclusion criteria for gas accumulation in previous studies, combined with the currently unknown effect of post-mortem decomposition on injectate spread, limits direct comparability (12, 18, 24). The alteration in injectate spread in animals with an increased level of gas due to putrefaction was not assessed in this study. Therefore, further studies are needed to determine how cadaveric decomposition influences the distribution of solutions in this type of cadaveric research. Investigating the effect of postmortem decomposition on injectate distribution could represent a valuable future line of research, improving standardization and comparability with live subjects.

The LBDR and MBDR are the nerves that provide sensation and motor function to the epaxial musculature, vertebral lamina, ligaments, facet joints and skin from the midline to the axillary line (30, 31). Staining of the LBDR and MBDR is one of the most commonly observed findings after an ESP injection and may explain the clinical analgesic effect of the ESP block reported for procedures involving these structures (8, 32). In the present study, both the LBDR and MBDR were consistently stained with similar results to previous studies in dogs, and both approaches producing comparable staining (4, 18). An important finding was the greater cranial extent of MBDR and LBDR staining, and of dye spread within the ESP, relative to their caudal extent using both approaches. This is consistent with previous reports and suggests a preferential cranial distribution of the injectate within the erector spinae plane (25). These findings may have clinical relevance, as they suggest that positioning the injection caudal to the surgical site could take advantage of the preferential cranial spread observed in the present study. However, this hypothesis should be confirmed in live animals, as cadaveric dye distribution may not fully reflect local anesthetic spread under physiological conditions.

Staining of the VBSN, the nerves that provide sensation and motor function to the thoracic wall, ribs, pleura and sternum, was also demonstrated in both groups (30, 31, 33, 34). This is consistent with cadaveric studies in dogs, cats, and horses and with human reports of VBSN staining and paravertebral spread (2, 9, 12, 15, 18, 35, 36). Nevertheless, other canine and porcine studies found staining confined to the dorsal rami (4, 24). Epidural spread was also observed in both groups, with one animal presenting a more extensive spread compared to the rest of the animals. This is similar to findings in some cadaveric studies in dogs and horses (9, 18, 25), but not with others in dogs or cats (4, 12, 24, 37). Staining of the VBSN and the epidural spread identified in this study supports the potential analgesic effect of the ESP block for surgical or painful procedures involving the thorax, and is consistent with previous reports in dogs undergoing thoracic procedures following ESP block (10, 38). In addition, injections in both groups resulted in staining of the sympathetic trunk, consistent with findings from some studies in humans, dogs, and cats (8, 12, 18, 39) but not with others in dogs (4, 24, 37). Spread to the sympathetic trunk following ESP injection at T9 may contribute to the analgesic effects of the technique for thoracic visceral pain (40). However, the clinical relevance of unilateral sympathetic trunk staining following ESP block remains to be determined. Lymphatic spread, previously reported at T12 in dogs (18), was absent here, possibly reflecting anatomical differences at the injection site (22, 23). This variability of the spread, aside from the staining of the MBDR and LBDR, mirrors that reported in human studies (41–43). Moreover, it indicates that the mechanisms underlying these spread patterns remain unclear, with discrepancies likely attributable to differences in needle tip placement, injection depth, injectate volume and speed, body condition score and size, age of the animal, and inter-individual fascial anatomy across studies. Further standardized research is needed to better understand these distributions. Nevertheless, these results, together with current evidence, support the potential analgesic effect of the ESP block for the epaxial musculature, skin, and vertebrae, while coverage of the thoracic wall, thoracic cavity and viscera, sternum, and extended epidural spread remains inconsistent (10, 11, 32, 38).

In contrast to the TV approach, the LNG technique was associated with a greater risk of unintentional injection over the rib lateral to the DLETP, even when performed by different operators, altering the pattern of injectate spread. This was not reported at T12 in previous canine studies (18); however, even though the transverse process at T9 is longer than at other thoracic vertebrae, the DLETP remains comparatively small, creating a narrow target area of injection. These findings suggest that the TV approach may offer advantages over the LNG technique when performed at vertebrae with a DLETP. With the LNG approach, minor lateral or medial probe movements can displace the DLETP from the imaging field, allowing the dorsal surface of the rib or transverse process to be mistaken for it. In contrast, the TV approach provides a cross-sectional view that simultaneously displays the vertebral lamina, transverse process, DLETP, costotransverse joint, and rib tubercle. Consequently, cranial or caudal probe displacement brings the intertransverse and intercostal muscles into view, reducing the risk of misplacement. Additionally, intramuscular injection in the LNG approach produces a lenticular separation of longitudinal muscle fibers that resembles ESP hydrodissection, whereas the circumferential spread pattern seen in the TV approach more clearly indicates misplacement (19–21). Operator experience and manual dexterity may also have contributed to the misplaced injections observed with the LNG approach. Although both operators were experienced and familiar with the LNG approach, differences in individual technique could have affected needle manipulation and the accuracy of injectate deposition. LNG injections performed outside the intended target area were not the only ones associated with unexpected spread patterns. A small number of correctly performed injections in both groups showed an atypical cranial shift in MBDR staining, with the first stained nerve root located more cranially than expected relative to the injection site. In one case, this pattern was mirrored contralaterally, suggesting that individual anatomical variation, rather than technique alone, may independently influence the craniocaudal distribution of the injectate within the ESP.

Morphological evaluation of the transverse process of the thoracic vertebrae revealed marked variation in transverse process and DLETP length, as well as inconsistency in DLETP presence across vertebral levels. The DLETP was consistently absent in the most caudal thoracic vertebrae, though the specific vertebral level of absence varied between cadavers. These findings suggest that the TV approach described in this study should be applied only at vertebrae with DLETP, while the TV approach described by Herrera-Linares et al. (18) is recommended where this structure is absent.

This study is not exempt from limitations. Post-mortem changes may influence the behavior and distribution of injected dye solutions, potentially differing from patterns observed in live animals. Several physiological processes present in live animals are absent in cadavers. These include blood and lymphatic flow, negative thoracic pressure, muscle tone, thoracic movement during breathing, and local anesthetic diffusion, all of which are normally present *in vivo* and may contribute to the overall behavior of injected substances within tissue. As these processes are absent in a post-mortem model, the spread patterns observed in this study should be interpreted with this limitation in mind, and further studies in live animals would be valuable to confirm these findings. Another limitation was the number of anesthetists performing the injections in each group. While two anesthetists performed the injections for the LNG approach, only one carried out all injections in the TV group. Using only one operator may introduce operator-dependent bias related to skill level or familiarity with the TV technique. However, only one individual possessed the necessary training to perform this novel technique. Therefore, a second operator was avoided to reduce the risk of inaccurate needle placement and ensure consistency.

The results of this cadaveric study demonstrated that the TV and LNG US-guided ESP approaches performed over the DLETP of the 9th thoracic vertebra were feasible for administering an ESP injection at vertebrae where this anatomical structure is present. Both techniques produced comparable multisegmental spread within the ESP, with a preferential cranial nerve dye and spread distribution. However, the TV approach was associated with a lower risk of injectate misplacement outside the desired target area and subsequent undesired lateral spread, compared with the LNG approach. This study also showed that the DLETP was smaller than the transverse process and was consistently identified in vertebrae cranial to T11, although it may also be present at T11. Therefore, the TV approach described in this study should not be replicated in vertebrae without a DLETP. Clinical studies are warranted to evaluate the analgesic efficacy and safety of this technique at thoracic vertebrae with a DLETP.

## Data Availability

The original contributions presented in the study are included in the article/Supplementary material, further inquiries can be directed to the corresponding author.

## Supplementary material

Supplementary video S1 Video illustrating the ultrasound (US) image of the novel US-guided transverse approach of the ESP block at T9. As the US probe is moved cranially, the rounded bony shadow of the rib appears on the lateral aspect of the image. The rib (R) is traced in a lateral-to-ventromedial direction until it contacts the hyperechoic surface of the corresponding transverse process (TP), which appears medially. Using an in-plane technique, the needle is advanced in a dorsolateral-to-ventromedial direction until the tip reaches the dorsal aspect of the dorsolateral edge of the transverse process (DLETP) after passing over the costotransverse joint (CTJ). D, dorsal; M, medial; L, lateral; P, pleura; V, ventral.
